# Medical guidelines for Li–Fraumeni syndrome 2019, version 1.1

**DOI:** 10.1007/s10147-021-02011-w

**Published:** 2021-10-11

**Authors:** Tadashi Kumamoto, Fumito Yamazaki, Yoshiko Nakano, Chieko Tamura, Shimon Tashiro, Hiroyoshi Hattori, Akira Nakagawara, Yukiko Tsunematsu

**Affiliations:** 1 Department of Pediatric Oncology, National Cancer Center Hospital, Tokyo, Japan; 2 Department of Pediatrics, Keio University School of Medicine, Tokyo, Japan; 3 Division of Brain Tumor Translational Research, National Cancer Center Research Institute, Tokyo, Japan; 4 Department of Pediatrics, The University of Tokyo Hospital, Tokyo, Japan; 5 Medical Information and Genetic Counseling Division, FMC Tokyo Clinic, Tokyo, Japan; 6 Department of Sociology, Graduate School of Arts and Letters, Tohoku University, Sendai, Japan; 7 Department of Clinical Genetics, National Hospital Organization Nagoya Medical Center, Aichi, Japan; 8 Saga International Heavy Ion Cancer Radiation Therapy Center, Saga, Japan

**Keywords:** Li-Fraumeni syndrome, Guideline, TP53, Chompret criteria, Surveillance

## Abstract

Li–Fraumeni syndrome (LFS) is a hereditary tumor that exhibits autosomal dominant inheritance. LFS develops in individuals with a pathogenic germline variant of the cancer-suppressor gene, *TP53* (individuals with *TP53* pathogenic variant). The number of individuals with *TP53* pathogenic variant among the general population is said to be 1 in 500 to 20,000. Meanwhile, it is found in 1.6% (median value, range of 0–6.7%) of patients with pediatric cancer and 0.2% of adult patients with cancer. LFS is diagnosed by the presence of germline *TP53* pathogenic variants. However, patients can still be diagnosed with LFS even in the absence of a *TP53* pathogenic variant if the familial history of cancers fit the classic LFS diagnostic criteria. It is recommended that *TP53* genetic testing be promptly performed if LFS is suspected. Chompret criteria are widely used for the *TP53* genetic test. However, as there are a certain number of cases of LFS that do not fit the criteria, if LFS is suspected, *TP53* genetic testing should be performed regardless of the criteria. The probability of individuals with *TP53* pathogenic variant developing cancer in their lifetime (penetrance) is 75% for men and almost 100% for women. The LFS core tumors (breast cancer, osteosarcoma, soft tissue sarcoma, brain tumor, and adrenocortical cancer) constitute the majority of cases; however, various types of cancers, such as hematological malignancy, epithelial cancer, and pediatric cancers, such as neuroblastoma, can also develop. Furthermore, approximately half of the cases develop simultaneous or metachronous multiple cancers. The types of *TP53* pathogenic variants and factors that modify the functions of *TP53* have an impact on the clinical presentation, although there are currently no definitive findings. There is currently no cancer preventive agent for individuals with *TP53* pathogenic variant. Surgical treatments, such as risk-reducing bilateral mastectomy warrant further investigation. Theoretically, exposure to radiation could induce the onset of secondary cancer; therefore, imaging and treatments that use radiation should be avoided as much as possible. As a method to follow-up LFS, routine cancer surveillance comprising whole-body MRI scan, brain MRI scan, breast MRI scan, and abdominal ultrasonography (US) should be performed immediately after the diagnosis. However, the effectiveness of this surveillance is unknown, and there are problems, such as adverse events associated with a high rate of false positives, overdiagnosis, and sedation used during imaging as well as negative psychological impact. The detection rate of cancer through cancer surveillance is extremely high. Many cases are detected at an early stage, and treatments are low intensity; thus, cancer surveillance could contribute to an improvement in QOL, or at least, a reduction in complications associated with treatment. With the widespread use of genomic medicine, the diagnosis of LFS is unavoidable, and a comprehensive medical care system for LFS is necessary. Therefore, clinical trials that verify the feasibility and effectiveness of the program, comprising LFS registry, genetic counseling, and cancer surveillance, need to be prepared.

## Introduction

Li–Fraumeni syndrome (LFS) is a hereditary cancer predisposition syndrome, in which multiple cancerous tumors are likely to develop over a person’s lifetime. While concerns have long surrounded this disease, underlying LFS is often overlooked, as a diverse range of cancers develop across a wide range of age groups. In the past, LFS was suspected from medical and family histories, and was diagnosed with a *TP53* genetic test. However, as cancer genomic medicine is becoming more widespread, an increasing number of cases are diagnosed as secondary findings of a cancer genetic panel test. However, no system has been established for following LFS in Japan, and there is no consistent approach in clinical settings.

Experts of hereditary tumors, including LFS, gathered in 2016 at the Childhood Cancer Predisposition Workshop, which was held as a subcommittee of American Association of Cancer Research (AACR). As a result, the optical surveillance and care standard for pediatric hereditary tumors was formulated based on precision genetics, and it was reported in 17 papers in the Clinical Cancer Research Journal in 2017 [1–17]. A comprehensive medical care system for hereditary tumors with onset in childhood and adolescents and young adults (AYA) needs to be established in Japan. Thus, in 2017, the Health Science and Labor Research Grants “A study to implement cancer genomic medicine system for hereditary tumors onset in childhood” Group was formed, with the preparation of LFS medical guidelines as the research objective.

## Basic items

### What is LFS?

LFS is a hereditary tumor syndrome caused by a pathogenic variant of *TP53*, a tumor-suppressor gene, which leads to a high probability of cancer during a lifetime. LFS was first reported by Frederic Li and Joseph Fraumeni in 1969, when cancer with a unique spectrum was observed in four families with a proband with rhabdomyosarcoma [18]. In 1988, Li and Fraumeni defined the classic LFS diagnostic criteria based on the analysis of 24 families, in which individuals inherited early-onset cancers through autosomal dominant inheritance (Table 1) [19]. In addition, in 1990, Malkin et al. showed that the causal gene of LFS is the *TP53* pathogenic variant [20].

**Table 1:** Classic LFS diagnostic criteria and Chompret criteria

*Classic LFS diagnostic criteria*
Meets all of the following The proband had an onset of sarcoma at < 45 years old The first-degree relatives developed cancer at < 45 years old The first- and second-degree relatives were diagnosed with cancer < 45 years old, or developed sarcoma, regardless of age
*Chompret criteria for TP53 screening*
[Family history] The proband developed an LFS core tumor (breast cancer, osteosarcoma, adrenocortical cancer, or brain tumor) < 46 years old At least one first- or second-degree relative had a history of an LFS core tumor < 56 years old If the proband has breast cancer, close relatives with breast cancer should be excluded [Multiple cancers] The proband has multiple cancers (excluding bilateral breast cancers), two of which are LFS core tumors that first developed < 46 years old [Rare cancers] Patients with adrenocortical cancer, choroid plexus cancer, and anaplastic rhabdomyosarcoma Family history is not applicable [Juvenile breast cancer] Breast cancer patients aged ≤ 31 years

### Epidemiology

In the general public, the ratio of individuals with germline *TP53* pathogenic variant (individuals with *TP5*3 pathogenic variant) is said to be 1 in 5000 or 1 in 20,000 [21, 22]. Recently, about 64,000 people were randomly extracted from three databases of the US National Cancer Institute, and 131 were found to be individuals with *TP5*3 pathogenic variant (0.2%, about 1 in 500) [23]. In Japan, 0.27% people in the database of Tohoku Medical Megabank Organization (2KJPN) were found to be individuals with *TP5*3 pathogenic variant [24].

The incidence of cancer patients with the *TP53* pathogenic variant has been reported by two large-scale studies on pediatric cancers [25, 26], and four clinical sequence studies on cancer [27–30]; both of which indicated that the incidence is about 2%. In adult patients with cancer, four of 1566 (0.2%) were found to be individuals with *TP5*3 pathogenic variants in the clinical sequence performed by the US Memorial Sloan Kettering Cancer Center [31].

### Diagnosis

#### Genetic diagnosis and classic LFS

The causal gene of LFS is *TP53*, and LFS is diagnosed by detecting *TP53* pathogenic variants [20]. However, some people meet the classic LFS diagnostic criteria without the pathogenic *TP53* variant, and such individuals are diagnosed with LFS [19].

#### Chompret criteria

The Chompret criteria are used to perform *TP53* genetic testing for suspected LFS (Table 1). As *TP53* was reported to be the causal gene of LFS, it was found that many patients with cancer had *TP5*3 pathogenic variant despite not satisfying the classic LFS diagnostic criteria; thus, the Chompret criteria was proposed to avoid any cases of LFS. The Chompret criteria consist of items on family history, multiple cancers, rare cancers, and juvenile breast cancer based on the clinical data of LFS; since it was first proposed in 2001 [32], it has been revised in 2009 [33] and 2015 [34]. The Chompret criteria will be revised as needed as the clinical presentation of LFS is further elucidated.

#### Explanation

In terms of “rare cancer” as an item of the Chompret criteria, the ratio of individuals with *TP5*3 pathogenic variant is high among patients with adrenocortical cancer and choroid plexus carcinoma, and its reproducibility in embryonal anaplastic rhabdomyosarcoma has not been reported. The ratio of individuals with *TP5*3 pathogenic variant among patients with adrenocortical cancer is 50–100% for those aged < 18 years (median of 75%) and 4–33% for those aged ≥ 18 years (median of 13%) [21, 25, 26, 35–41]; thus, in adrenocortical cancer, LFS is suspected, regardless of the patient’s age. The ratio of individuals with *TP5*3 pathogenic variant among patients with choroid plexus carcinoma was 25–100% (median of 45%), in which all patients aged < 18 years [21, 25, 42–45]. Furthermore, 11 of 15 embryonal anaplastic rhabdomyosarcoma cases (73%) were pathogenic *TP53* variant carriers [46]; however, as there is only one report, further investigation is needed.

In terms of the Chompret criteria item “juvenile breast cancer,” the ratio of individuals with *TP5*3 pathogenic variant in patients with normal breast cancer patients, breast cancer patients with premenopausal onset, and breast cancer patients with a family history of breast cancer was 0–1.0% [47–52], 0–3.8% [50, 53–57], and 1.0–2.9% [58, 59], respectively. In contrast, the ratios of individuals with *TP5*3 pathogenic variant among patients with breast cancer aged < 31, 31–40, 41–50, and ≥ 51 years were 0–3.8%, 0–2.6%, 0–0.8%, and 0–0.2%, respectively. The ratio of individuals with *TP5*3 pathogenic variant is higher among breast cancer patients with premenopausal onset or family history of breast cancer than in those with breast cancer; however, there is no clear basis for considering 31 years as a cut-off.

#### Secondary findings

As genomic medicine has become more widespread, *TP53* pathogenic variants are occasionally detected as a secondary finding of genetic panel testing. When *TP53* pathogenic variants are detected in an analysis of somatic line, many such pathogenic variants are acquired changes and do not exist in the germline, but some might; therefore, germline testing is necessary. Upon carefully determining the likelihood of finding the same pathogenic variants from somatic line analysis in the germline based on medical and family history, germline analysis should be performed as needed.

Please refer to “Recommendations for communication process in genomic medicine—part 1: cancer genetic panel test (2nd edition)” (Japan Agency for Medical Research and Development (AMED) Genomic Drug Promotion Research Project, research supervisor: Shinji Kosugi, https://www.amed.go.jp/content/000056785.pdf) for the interpretation of secondary findings from cancer genetic panel tests.

### Clinical presentation

#### Cancer penetrance and LFS core tumors

Individuals with *TP5*3 pathogenic variant have a high probability of developing cancer during their lifetime (penetrance) (75% for men and almost 100% for women) [34, 60]. Juvenile onset of cancer and multiple onsets and types of cancers in one patient are characteristics of LFS. Breast cancer, osteosarcoma, soft tissue sarcoma, brain tumor, and adrenocortical cancer are LFS core cancers, which have high incidence in patients with LFS. As mentioned earlier, breast cancer often has premenopausal onset, rhabdomyosarcoma is a common soft tissue sarcoma in children, and leiomyosarcoma is common in adults. Common brain tumors include choroid plexus carcinoma with early onset in infancy to childhood, medulloblastoma, and glioma. LFS is known to lead to the onset of various types of cancers including epithelial carcinoma, such as gastric cancer and colorectal cancer; hematological malignancies, such as leukemia and lymphoma; and pediatric cancers, such as neuroblastoma.

As the Chompret criteria state, patients with adrenocortical cancer, choroid plexus carcinoma, and anaplastic rhabdomyosarcoma have *TP53* pathogenic variants at high probability. In recent years, it has been shown that sonic hedgehog (SHH) medulloblastoma and hypodiploid acute lymphocytic leukemia are most likely associated with LFS.

#### Explanations

Cancer penetrance: The cancer penetrance of individuals with *TP5*3 pathogenic variant was 73.8% over an average follow-up period of 28 years [60]. Compared to the general population, SIR was 41.1 (95% CI: 29.9–55.0) [61] and RR was 4.0 (95% CI: 3.3–4.8) [62]; thus, carcinogenesis risk is significantly higher for individuals with *TP5*3 pathogenic variant. Within the same family, the carcinogenesis risk of pathogenic *TP53* variant carriers is higher; where RR was 43.8 (95% CI: 18.5–103.5) and 18.5 (95% CI: 8.3–41.3) [63] for women and men, respectively. Multivariate analysis indicated that the OR was 1,075 (95% CI: 358–3299) and 151 (95% CI: 60–380) [64], respectively.

Table 2 shows the comparison of LFS core cancer penetrance for individuals with *TP5*3 pathogenic variant and the general population. When osteosarcomas are comprehensively analyzed, compared to general population, the OR was 1.69 (95% CI: 1.01–2.80) [65], while the HR was 15.7 (*P* < 0.0001) within the family [66]; thus, the penetrance of osteosarcoma is significantly higher for individuals with *TP5*3 pathogenic variant. Furthermore, in southeastern Brazil, there is a high incidence of LFS with *TP53* p.R337H, and the risk of adrenocortical cancer in this area is RR of 2047 (95% CI: 455–9212) compared to the general population [67]. The precision of adrenocortical cancer onset prediction is reported to have a sensitivity of 84.6% (95% CI: 54.6–98.1) and specificity of 99.7% (95% CI: 99.7–99.8%).

**Table 2:** Comparison of penetrance for LFS-related cancer patients and the general public

	Penetrance (%)	Comparison with general public	
		SIR (95%CI)	RR (95%CI)
All cancers	73.8	41.1 (29.9–55.0)	4.0 (3.3–4.8)
Core cancers			
Breast cancer	25.0–59.6	105.1 (55.9–179.8)	6.4 (4.3–9.3)
Osteosarcoma	6.3–15.5	289.0 (93.1–674.4)	107 (49–203)
Soft tissue sarcoma	14.3–26.7	302.8 (130.4–596.8)	61 (33–102)
Brain tumor	5.4–13.0	45.0 (9.0–131.5)	35 (19–60)
Adrenocortical carcinoma	1.7–13.0		2047 (455–9212)^a^

*SIR* standardized incidence ratio; *RR* relative risk

^a^Subject is *TP53* p.R337H

As for tumors other than LFS core cancers, individuals with *TP5*3 pathogenic variant develop various types of cancers, such as gastrointestinal cancer, urogenital cancer, hematological malignancy, lung cancer, and malignant melanoma. But evidence suggesting that penetrance of these cancers is higher than those of the general public is limited [34, 60–62, 68].

#### Explanation

*Age of cancer onset*: An analysis of 415 individuals with *TP5*3 pathogenic variant in 214 French families with LFS showed that the cancer penetrance for young people aged 0, 5, and 18 years were 4%, 22%, and 41%, respectively [34]. Furthermore, an analysis by the US National Cancer Institute of 286 pathogenic *TP53* variants in 107 families with LFS showed that the 50% cumulative cancer onset age was 46 years for men and 31 years for women. An analysis of 145 individuals with *TP5*3 pathogenic variant from 10 families by the University of Texas MD Anderson Cancer Center (MDACC) showed that when subjects were divided into young and old to maximize the relative risk for probands/parents/grandparents, the carcinogenesis risk of young individuals with *TP5*3 pathogenic variant was 133- and 165-fold higher than that of the general public for men and women, respectively (older pathogenic *TP53* variant carriers had 15- and 26-fold higher risks for men and women, respectively) [66].

#### Pathogenic *TP53* variant genotype and phenotype and factors that influence clinical presentation

More than 250 types of *TP53* pathogenic variants (pathogenic/likely pathogenic variant) have been reported; however, as many of *TP53* variants are missense variants, the interpretation of pathological significance can be challenging and must be carefully interpreted by experts. In addition, to avoid different interpretations of pathological significance between health care providers and facilities, an inspection agency that performs interpretation of pathological significance based on the standardization, such as the US ACMG, or consultation by experts familiar with interpretation of pathogenic *TP53* variants should be utilized.

About 70% of pathogenic *TP53* variants are missense variants [35, 69], and abnormalities in splicing, intragenic deletions, frameshift variants, nonsense variants, in-frame insertions/deletions, and intron deletion have been reported [35]. There are six hotspots for pathogenic *TP53* variants (p.R175H, p.G245S, p.R248Q, p.R248W, p.R273H, and p.R282W), and about 20% of LFS families have some hotspots for pathogenic germline variants [35]. The types of germline pathogenic variants are similar to pathogenic variants of somatic line for cancer that occurs in non-carriers of pathogenic *TP53* variants. However, even for an abnormality in the same location, the resulting cancers are likely to vary widely [70]. Individuals with missense variants in the DNA binding site for *TP53* tend to develop cancers with relatively high-grade malignancy, whereas carriers of missense variants in the non-DNA binding site tend to develop cancers with relatively low-grade malignancy [34, 71]. At present, the clinical presentation cannot be predicted from the genotype. Meanwhile, factors that modify functions of *TP53* might influence the type of cancer and the age of onset.

#### Explanation

There are significant differences in the frequency at which individuals with missense variants in the DNA binding site of *TP53* DNA develop rhabdomyosarcoma and osteosarcoma [65]. Moreover, the frequency of leiomyosarcoma is high when there are changes in tetramerization domains, such as *TP53* codon 337 or 344 [65]; however, reproducibility has not been confirmed. In contrast, carriers of *TP53* p.R337H, present at higher frequency in the southeastern part of Brazil have a high incidence of adrenocortical cancer. As for the age of cancer onset, individuals with missense variants, especially individuals with missense variants in the DNA binding site, tend to exhibit earlier onset of cancer; however, the reproducibility of such findings has not been demonstrated [34].

There are several reports on the involvement of a single-nucleotide polymorphism (SNP: rs2279744, SNP309) of *MDM2*, which regulates the degradation of p53, with juvenile onset of soft tissue sarcoma and breast cancer as well as several other cancers [72]. Indeed, in individuals with *TP5*3 pathogenic variant, the age of cancer onset is significantly younger when the *TP53* codon 72 has the Arg allele compared to individuals in which the same site exhibits a homozygous Pro allele. In cases exhibiting *MDM2* SNP309 G allele, and *TP53* codon 72 Arg allele in particular, the age of onset is clearly younger than when *MDM2* SNP309 T and *TP53* codon 72 Pro allele are homozygous [73]. In addition, it has been reported that polymorphism of *TP53* intron 3 (PIN3) invokes juvenile onset of cancer [74, 75]. Patients with cancer have short telomere lengths, and the speed of telomere shortening is fast for individuals with *TP5*3 pathogenic variant [76], and hypermethylation of miR-34A lowers the survival rate [77–79]. However, these studies have not been reproduced. Factors that modify functions of *TP53* are related to the clinical presentation of LFS; therefore, basic medical research, such as multigenic analysis, is necessary.

### Prevention and treatment

Clinical trials of chemoprophylaxis, such as metformin, are in progress as cancer preventive agents for pathogenic *TP53* variant carriers, yet no drug has been confirmed to be effective. Therefore, in addition to usual cancer preventive measures, such as avoiding alcohol, smoking, and exposure to ultraviolet rays, radiation, and carcinogenic substances, the main measures are risk-reducing surgical treatment of LFS target organs, such as the breasts, and early detection and treatment through cancer surveillance. While clinical trials of molecular targeted drugs targeting the *TP53* and *TP53* pathway are in progress, their efficacy has not yet been proven. Although it has been reported that *TP53* pathogenic variants are factors of poor prognosis for B-cell acute lymphoblastic leukemia [80], evidence of resistance of LFS-associated cancers to treatment are limited; thus, standard treatment of each cancer is prioritized. To avoid the onset of secondary cancer, it is recommended to avoid radiation exposure, irradiation, and alkylating agents as much as possible. However, such treatment methods will be used in cases where it is considered unavoidable, such as rhabdomyosarcoma [6]. One characteristic of LFS is multiple cancers, and surgical treatment during an early stage of the disease will avoid sequela of multiple treatments. From this perspective, early detection through surveillance is important.

### Cancer surveillance

#### 1.6.1 Cancer surveillance and protocol

Cancer surveillance for individuals with *TP5*3 pathogenic variant began with the “Toronto Protocol” implemented in Canada and the USA [81, 82]. As LFS-associated cancers are diverse and affect whole body, whole-body (WB) MRI, brain MRI, breast MRI (adult women only), ultrasonography (US), and endoscopy (adult only) are performed on a tight schedule. The results of the Toronto Protocol were reported in 2011 and 2016, and indicated that surveillance improves the survival rate of individuals with *TP5*3 pathogenic variant. Subsequently, various countries initiated cancer surveillance programs, and at present, six countries are using 12 cancer surveillance programs [83]. The US National Comprehensive Cancer Network (NCCN) guideline also recommends similar cancer surveillance (NCCN Clinical Practice Guidelines in Oncology, Genetic/Familial High-Risk Assessment: Breast, Ovarian, and Pancreatic. Ver 1. 2020–December 4, 2019 can be downloaded from the NCCN website). As LFS has high cancer penetrance and often presents as multiple cancers, the detection rate of such surveillance is high, and increases in proportion to the follow-up period.

In 2016, a subcommittee of AACR, Childhood Cancer Predisposition Workshop, gathered experts of various fields from around the world, and formulated the standard for appropriate surveillance and care of hereditary tumors with childhood onset, including LFS. The results were published in 17 papers on Clinical Cancer Research in 2017 [6]. The recommended cancer surveillance method used the Toronto Protocol as the basic framework, and this protocol can be used a template for future surveillance protocols (Table 3).

**Table 3:** Recommended surveillance of LFS

*Children (birth to 17 years old)*
[General evaluation] Full check-up every 3 to 4 months, including blood pressure, growth curve (with particular attention to a rapid increase in height and weight), Cushing-like facial features, masculinization (pubic hair, armpit sweating, adult body odor, male-pattern baldness, labial hypertrophy, penile growth), and neurological assessment Cooperation with attending physician [Adrenocortical carcinoma] Abdominal and pelvic ultrasound every 3–4 months If ultrasound is not possible, blood test^a, b^ every 3–4 months to measure total testosterone, dehydroepiandrosterone, androstenedione [Brain tumor] Brain MRI performed every year, the first of which is contrast MRI. Subsequently, contrast is not necessary as long as the previous MRI is normal and no new abnormalities are confirmed [Bone and soft tissue tumor] Whole-body MRI^c^ every year
*Adults [from 18 years old]*
[General evaluation] Full physical check-up every 6 months All medical phenomena promptly evaluated by the attending physician [Breast cancer] Examine breasts: from 18 years Breast exam twice a year from 20 years Breast MRI^d^ every year from 20 to 75 years Risk-reducing mastectomy should be considered [Brain tumor] Brain MRI^a^ performed every year, the first of which is contrast MRI. Subsequently, contrast is not necessary as long as the previous MRI is normal, and no new abnormalities are confirmed [Bone and soft tissue tumor] from 18 years old Whole-body MRI^c,d^ every year Abdominal and pelvic ultrasound every 12 months [Gastrointestinal cancer] from 25 years old Upper and lower GI endoscopy every 2 to 5 years [Malignant melanoma] from 18 Dermatological examination every year

^a^Always sample blood at the same time of the day and at the same laboratory

^b^Utility of biopsy to detect adrenocortical carcinoma is not stipulated

^c^Whole-body MRI is performed from head to toes, including all limbs

^d^Breast MRI and abdominal and pelvic ultrasound are alternately performed with whole-body MRI (at least 1 examination every 6 months)

#### Effectiveness and disadvantages of surveillance

The absolute indexes that show the efficacy of the surveillance are “decrease in the mortality rate of cancer” and “decrease in the incidence of advanced cancer,” while the relative index is shown as “sensitivity and specificity”. However, efficacy has not been verified, and the disadvantages include false positives, overdiagnosis, adverse events associated with the sedation used for the imaging, and psychological impact.

#### Explanation

In terms of efficacy of surveillance, the Toronto Protocol with the longest follow-up period divided individuals with *TP5*3 pathogenic variant into the surveillance group (*n* = 40) and the non-surveillance group (*n* = 49) [82]. According to the report, transition from the non-surveillance group to the surveillance group was permitted, but according to the intention to treat analysis, the detection rate of cancer was notably lower in the surveillance group (17.5% vs 87.8%, relative risk of 0.30, 95% CI 0.17–0.56). Therefore, the relative risk of cancer-related death in the surveillance group was 0.11 (95% CI 0.03–0.45); thus, further follow-up survey might be necessary to prove the efficacy of the surveillance.

In the UK, hereditary breast cancer surveillance is conducted in high-risk subjects, such as individuals with *BRCA1/2* or *TP53* pathogenic variants [84]. This method assessed the sensitivity and specificity, as the alternative index of efficacy over a short period of time, and it was used as the national program. Moreover, it is becoming established as a surveillance method that can be used to suppress disadvantages as much as possible by limiting target organs, target ages, and sex (women only).

#### Explanation

The disadvantages of surveillance are shown below.

*False positive*: Calculation of accurate numbers was difficult, but as an alternative index, the positive rate was about 20–30% for WB MRI (Table 4) [82, 85–89]. In the meta-analysis of WB MRI discussed below, 78.7% of positive subjects had no abnormalities or had benign tumors [83]. Four studies that used MRI, US, and multiple other tests for surveillance were summarized [81, 82, 85–87], and when brain MRI, breast MRI, and abdominal ultrasound were added to 59.4% of WB MRIs overall, about 90% of cancers could be detected. However, it should be noted that the number of false positives also increases in proportion to the number of test items.

**Table 4:** Positive rate of surveillance and cancer detection in international studies

	Toronto	NCI	MDACC	DFCI	SIGNIFY	Brazil	Meta-analysis
*N*	59	116	53/35	20	44	59	578
Test items	Multiple tests including WB-MRI	Multiple tests including WB-MRI	WB-MRI/brain MRI	WB-MRI and blood tests	WB-MRI	WB-MRI	WB-MRI
Follow-up period	Median of 32 months (12–87 months)	Median of 3.8 years (6 months to 54 years)	Median of 16 months (5.5 to 24.5 months)	Median of 3 years (1 month to 4 years)	Unknown	Maximum of 55 months	
Positive rate							
WB-MRI	Unknown	27.5% baseline	58.5% baseline	37.8% cumulative	36.4% baseline	11.8% baseline, 6.7% second test	29.9% baseline
Other	Unknown	Unknown	28.6% in baseline brain MRI	0% blood tests			
Cancer detection rate	13.6% cumulative	4.3% baseline	15.1% baseline WB-MRI, 8.6% baseline brain MRI	2.2% cumulative	9.1% baseline	3.4% baseline, 1.7% second test	6.7% baseline

*NCI* National Cancer Institute; *MDA* MD Anderson Cancer Center; *DFCI* Dana-Faber Cancer Institute; *WB* whole body

*Overdiagnosis*: Analysis of the above four studies revealed that 40.6% of the detected tumors were low-grade lesions, and a high percentage was noted in children. For pediatric cases, even if the tumor is low-grade, treatment may follow the protocol used for high-grade tumors. This cannot be simply referred to as overdiagnosis. Thyroid cancer and non-invasive breast cancer have been shown to be detected in adult cases. It is predicted that surveillance will increase the rate of overdiagnosis.

*Adverse events associated with sedation used for imaging*: Adverse respiratory and cardiovascular events are predicted, but no previous study has targeted individuals with *TP5*3 pathogenic variant.

Psychological impact: described in “Psychological issues” section.

#### Precision of tests used for surveillance

LFS has high cancer penetrance and often leads to the development of multiple cancers. Therefore, the cancer detection rate is high with various tests, in particular, during the baseline screening. Though the detection rate decreases in the subsequent screenings, a certain level of cancer detection rate is likely maintained. Although the false positive rate is also high, it could be reduced by comparing findings to previous images, as the radiologists become more familiar with imaging and as the number of screenings increases.

#### Explanation

In a Dutch multi-facility study that conducted breast cancer surveillance in people with high risk of breast cancer, such as individuals with *TP53* and *BRCA1/2* pathogenic variant (MRISC, Dutch MRI Screening) [90], the breast cancer detection rate was high at 9.6% and 12.1% at the baseline and second MRI, respectively, and it remained high in the subsequent screenings: 6.7%, 3.4%, and 4.1%. In contrast, the positive rate (rate of subjects requiring further tests), the alternative index of false positive rate, was around 12% from the baseline to the third screenings, respectively, which decreased to 9.3% and 6.9% in the fourth and fifth screenings. Similar trends were found in cancer surveillance on LFS; thus, while maintaining a certain level of cancer detection, false positives, a disadvantage of surveillance, are expected to decrease as the number of screenings increases. We discussed the precision of each screening method below.

*WB MRI*: WB MRI is performed with shortened imaging time. Although the image resolution is low, the cancer detection rate is high compared to other testing methods. When limited to the first WB MRI, the cancer detection rate was 3.6–13.6% [82, 85–89]. In contrast, WB MRI detects many benign lesions. In a British SIGNFY study, healthy individuals were used as a control group to individuals with *TP53* pathogenic variant, but in the baseline WB MRI, benign lesion was discovered in seven out of 44 healthy subjects (15.9%) [89]. According to the SIGNIFY study and US MDACC study [85], while it was difficult to determine the specificity of WB MRI, the sensitivity was 70%.

A meta-analysis reported the results of baseline WB MRI from 12 studies [83]. Among the 572 individuals with *TP53* pathogenic variant, 173 were determined to require further tests (positive rate of 29.9%). A total of 35 localized new cancers were detected in 34 subjects (cancer detection rate of 5.9% and positive predictive value of 19.7%). All subjects were given curative treatments. The breakdown of false positive cases were as follows: 119 subjects with no abnormalities (68.8%), 19 with benign tumors (9.8%), and seven with recurrence or metastasis of existing cancer (4.0%). There are few records of false negative cases, and the sensitivity and specificity were not calculated. Although the positive rate was high, as discussed above, it might be decreased by comparing findings with previous images, as radiologists become more familiar with the technique, and as the duration of surveillance accumulates [90].

*Brain MRI*: Brain tumor detection cannot be substituted by WB MRI. Among three studies in which brain MRI was used [82, 85, 86], one was the Toronto Protocol, in which the cumulative cancer detection rate based on brain MRI was 13.6% (median follow-up period, 32 months; range, 12–87 months). If we limit to the baseline brain MRI, the positive rate was 4.3% (5/116) and 22.9% (10/35), and the cancer detection rate was 1.7% (2/116) and 8.6% (3/35). The report by MDACC calculated a sensitivity of 60% and specificity of 80%.

*Breast MRI*: There are three reports, and the Toronto Protocol did not detect breast cancer (median follow-up period, 32 months; range, 12–87 months) [82]. According to the report by the US National Cancer Institute, the breast cancer detection rate on baseline MRI was 9.1% (2/22) [86]. In UK, breast cancer surveillance in those with high risk of breast cancer was conducted (MARIBS study), wherein the breast cancer detection rate on baseline MRI was 5.6%, and the cumulative cancer detection rate was 11.1% (follow-up period, 52–120 months) [84]. Although the positive rate could not be identified, benign lesions were detected. No previous reports have detailed the sensitivity and specificity; therefore, further investigation is necessary.

*Abdominal US*: In southeastern Brazil where the number of individuals with *TP53* pathogenic variant is high, *TP53* genetic testing was performed in about 180,000 newborns and close relatives. Individuals with *TP53* pathogenic variant were divided into the surveillance (*n* = 346) and non-surveillance groups (*n* = 391), wherein the former received routine abdominal US [67]. There were seven and eight subjects with adrenocortical cancer in the surveillance group and the non-surveillance group, respectively; the relative risk was 0.989 (95% CI 0.362–2.699), and showed no significant difference. As a result of the preemptive effect of the surveillance, all adrenocortical cancer cases detected in the surveillance group were stage I and could be treated with surgical resection only. In contrast, in the non-surveillance group, advanced cancer cases were common, which in addition to surgical treatment, required chemotherapy, and mortality was observed one. In the surveillance group, tumors, such as neuroblastoma, were detected; therefore, long-term follow-up may be able to reduce the incidence and mortality of advanced abdominal cancer.

*PET/CT* Radiation on individuals with *TP53* pathogenic variant is not recommended because it could cause secondary cancer; however, there are two case reports [91, 92], in which, the positive rates were 20.0% (6/30) and 33.3% (5/15), and the cancer detection rates were 10% (3/30) and 20% (3/15).

### Psychological issues

Patients and their family members experience various emotions and thoughts as a result of discussions regarding suspected LFS, *TP53* genetic testing, and treatment and surveillance for prevention after being diagnosed with LFS. Some might hope for a negative genetic test result, or some might wish for no negative findings in surveillance. Patients may be anxious about bad results, although some may have positive attitudes. Moreover, patients may want to know the situation, whether good or bad, to work on prevention, or want to be useful for their family members. Despair and fear, the psychological burden of talking to their family, and many other emotions are often described. Such psychological situations vary depending on whether the person has cancer or not, type of cancer they have or family has, the treatment conditions, the duration since diagnosis, age, sex, personality, or if the person being tested is themselves or their child. Furthermore, factors that modify such thoughts and emotions include medical conditions, cost, concern of the impact on life insurance, financial situation, school and work, marriage and child-rearing, and living with family.

It is difficult for others to understand the complex thoughts and emotions of each person, but it is significant in terms of psychological support for health care providers to offer opportunities to listen and show empathy and understanding. It is important to understand that it is natural for people facing serious health situations to feel anxiety, concerns, grief, anger, despair, and anguish, and to confirm these emotions without denying them. While most people are able to face the facts and deal with their emotions over time, it takes time to psychologically adapt to situations. Thus, rather than trying to quickly ease or resolve anxieties, health care providers should try to understand the patient’s psychological adaptation, listen to them without denying their feelings, and calmly be there for them. In addition, healthcare providers should accurately explain the latest information about LFS to subjects and family as many times as it takes, and carefully answer patients’ questions, as this will be a major support, and help them to calmly face the situation.

## LFS treatment guidelines

### (CQ1) Are the Chompret criteria useful as criteria to perform TP53 genetic testing for suspected LFS?

#### Recommendations

The Chompret criteria allow the detection of individuals with *TP5*3 pathogenic variant; however, as there are some patients with LFS who do not meet this criteria, it is not necessary to strictly adhere to this criteria when LFS is suspected (consensus rate* of 96%, 22/23).

* Consensus rates were determined by the votes of the 23 members of the study group, as described in the “Recommendation” paragraph of “Preparation method of the present treatment guidelines”.

#### Explanation

There is one report on the latest Chompret criteria (2015 version) [93], where the sensitivity was 75% (probability of not missing individuals with *TP5*3 pathogenic variant), and the specificity was not very high at 64.5% (probability to accurately diagnose subjects who are not carriers of *TP5*3 pathogenic variant). However, as it is important not to miss any individual with *TP5*3 pathogenic variant when diagnosing LFS, the Chompret criteria are useful for identifying individuals with *TP5*3 pathogenic variant. However, there is a certain number of individuals with *TP5*3 pathogenic variant who do not meet the Chompret criteria (false negative rate of 25%). For pediatric patients with cancer, family members are also often young and therefore might not still have developed cancer, which makes it difficult to obtain an accurate family history. It has been reported that 25% of cases are de novo LFS; therefore, when LFS is suspected from a history of juvenile onset of cancer, family history, or multiple cancer, *TP53* genetic testing is performed. While the classic LFS has high specificity (91.0–98.1%), its sensitivity is low (about 25.0–40.0%) [21, 62, 93].

Although *TP53* genetic testing is not covered by health insurance in Japan, it can be performed by laboratories at the patient’s own expense.

### (CQ2) Timing for implementing TP53 genetic testing

#### Recommendations

*TP53* genetic testing is performed immediately when LFS is suspected. However, there are various ethical, legal, and social issues (ELSI) surrounding patients with LFS, and the test should only be performed after the subject and family have carefully considered the situation (consensus rate of 96%, 22/23).

#### Explanation

AACR recommends that *TP53* genetic testing should be performed promptly when LFS is suspected [6], but when performing the test, it must be explained to subjects during genetic counseling that a diagnosis of LFS will have an effect on families and relatives, genetic testing and subsequent cancer surveillance are not covered by health insurance in Japan, and there are various ELSI (Table 5). In addition, it should be explained that missense variants are common in *TP53* germline variants, which could make the interpretation of pathological significance difficult.

**Table 5:** Ethical, legal, and psychological issues

*Issues derived from the nature of genetic information*
Genomic research and the protection of human rights: It is against basic human rights to examine the genetic properties of an individual without their consent Genetic information prior to onset: The right to know and the right not to know The right to access genetic information: The rights to privacy and confidentiality Sharing of genetic information with family: (1) Family members should not be coerced into undergoing testing—their autonomy must be respected (2) In addition to the anxiety of subjects with positive results, consideration must be given to the survivor’s guilt that subjects experience regarding negative results (3) Necessity to warn family members of the risk and requirements to disclose genetic information to the client without permission (requirements for the release of consfidentiality) Ethical issues of passing the gene onto children: (1) Reproductive decision making (2) Disclosure to the subject’s partner (3) Technical and ethical issues with prenatal examination and preimplantation diagnosis
*Testing children and those without the ability to consent*
Judging the necessity for early diagnosis: childhood onset and tests with established significance Necessity of explanation and support for children Responsibility of guardians, and support for the whole family Clear statement that children have rights to access the result of tests performed on them with permission provided by a legal representative when they reach the age of consent
*Prejudice and discrimination of genetic disease*
Social discrimination: marriage and employment Poor self-image and being spoiled by overprotective parents (fragile child syndrome)
*Storage and use of DNA and genetic information*
Registration of medical and family history, manner of follow-up and shared use of the gene bank Third-party access and data protection Ownership of materials and other research use Accumulation of the test result and record of the natural history
*Efficacy of the test, and ethics of safety*
Precision and specificity of the test Standard description of the test result, especially in relation to variants [5] Limit to the efficacy of prevention, treatment, and surveillance that suits the test result Cost of the test, treatment, and surveillance Uncertainty surrounding onset risk assessment, and psychological anxiety caused by its complexity Discrimination caused by leakage of the test result: Employment and insurance
*Health policy recommendations*
Examination of the quality assurance system at facilities that conduct genetic tests Risk division for secondary prevention of cancer: A study on the cost reduction effect of performing cancer examination of patients at high risk of hereditary diseases separately from members of the general public Primary prevention study of patients at risk of hereditary diseases: Instruction regarding lifestyle improvements, and clinical trial on primary prevention through chemical prevention Various problems associated with long-term psychological and social surveys Future direction of total genomic examination at general resident level, and examination of ethical issues

It has been indicated that treating LFS with radiation therapy and alkylating agents can damage DNA and increase the risk of secondary cancer onset. Therefore, when LFS is suspected in patients with cancer, it is highly significant to determine whether the subject has the germline *TP53* pathogenic variant [6].

LFS has a peak of onset for adrenocortical cancer and choroid plexus carcinoma in infancy [35, 94]. Specifically, the latter, which is a brain tumor, can cause severe sequalae if diagnosis is delayed. Therefore, when individuals with *TP53* pathogenic variant or their spouse is delivering a baby, performing the genetic test immediately after the delivery and knowing whether the child has LFS or not will lead to countermeasures for cancers that develop during infancy.

### (CQ3) Should radiation exposure and irradiation of individuals with TP53 pathogenic variant be avoided?

#### Recommendations

Epidemiological reports on increased incidence of secondary cancers in individuals with *TP53* pathogenic variant due to radiation exposure and irradiation are limited. However, theoretically, it is possible that cancer can develop; and thus, radiation should be avoided if there are other options (consensus rate of 91%, 21/23).

#### Explanation

*TP53* is induced in response to cellular stress that causes damage to DNA. *TP53* is called “the guardian of the genome,” and is at the center of the pathway that guides DNA repair, growth arrest, senescence, and apoptosis [95]. Therefore, theoretically, cells with weakened or disappeared *TP53* function have aberrant pathways, which may induce carcinogenesis. While reports describing secondary cancer being induced by radiation and irradiation in pathogenic *TP53* variant carriers are limited, it is recommended that radiation exposure from imaging tests, such as CT and PET-CT, and for treatment should be avoided as much as possible [6]. However, as there may be no other option for routine treatment, irradiation is allowed if the risk benefit balance indicates its utility.

Among individuals with *TP53* pathogenic variant who developed choroid plexus carcinoma, three out of 11 who received radiation therapy developed a different cancer, while only one out of 17 who did not receive radiation therapy developed cancer [96]. Cancers that developed following radiation therapy presented as hematological malignancies, and solid tumors in locations that were not irradiated. At the US St. Jude Children’s Hospital, 3006 patients who underwent whole-genome sequencing 5 years after the primary cancer were analyzed [97]. Patients with hereditary tumor with germline pathogenic variants of at least one of 60 cancer susceptibility genes, including *TP53,* had a significantly higher risk of sarcoma onset due to radiation therapy than non-carriers (breast cancer RR 13.9; 95% CI 6.0–32.2, sarcoma RR 10.6; 95% CI 4.3–26.3). However, in this report, 175 patients were diagnosed with hereditary tumors but only 10 were individuals with *TP53* pathogenic variant. The impact of radiation therapy on individuals with *TP53* pathogenic variant was not analyzed by category.

### (CQ4) Should cancer surveillance be conducted on individuals with TP53 pathogenic variant?

#### Recommendations

Cancer surveillance is recommended. However, the efficacy of cancer surveillance is still being studied, and it should be presented to subjects as one of the options (consensus rate of 96%, 22/23).

#### Explanation

Most cancer surveillances in progress overseas have short follow-up periods, and LFS is a rare disease with small number of subjects. Thus, the efficacy of cancer surveillance (reduced mortality and incidence of advanced cancer) has not yet been verified [67, 82, 83, 85–89]. However, most cancers detected by cancer surveillance are localized, and curative therapy is performed [83]. With a reduction in the therapeutic intensity, complications associated with therapy will be reduced and can lead to improved OQL. WB MRI used for cancer surveillance can only be performed a limited number of facilities in Japan, the false positive rate is high, and overdiagnosis is common. Moreover, in infancy, there are many disadvantages such as adverse events [98–112] associated with sedation (see “Effectiveness and disadvantage of surveillance” and “Precision of tests used for surveillance” sections). Not all psychological impacts of cancer surveillance on subjects are positive (see “Psychological issues”) [82, 88, 113–116]. Thus, it is recommended cancer surveillance should be suggested as an option and implemented in accordance with the wishes of the patient and their family. At this time, surveillance should be implemented on a clinical trial basis as much as possible, and nationwide data should be collected to promote the establishment of a standard surveillance program.

### (CQ5) Who are subjects of cancer surveillance?

#### Recommendations

Individuals with germline *TP53* pathogenic variant and those who meet the classic LFS diagnostic criteria (consensus rate of 100%, 23/23).

#### Explanation

In the Toronto Protocol, regardless of the onset, all individuals with *TP53* pathogenic variant are considered subjects [81, 82]; however, according to the recommended protocol of AACR (the AACR protocol), subjects are individuals with *TP53* pathogenic variant without cancer onset, and those who meet the classic LFS diagnostic criteria even if they do not have *TP53* pathogenic varients [6]. Families of the latter group also become subjects after taking the *TP53* genetic test. Many protocols target those who are in remission for a certain period of time following a cancer treatment, in addition to those without cancer onset [85–89]. This is because detection of recurrence and metastasis from existing onset has an impact on the determination of the cancer surveillance effect. However, since it has been indicated that about half of patients with LFS develop cancer within 5 years [34], it is valid to conduct surveillance even during cancer treatment or immediately after such treatment. In clinical trials, these cases need to be categories for analysis.

### (CQ6) When should the cancer surveillance of individuals with TP53 pathogenic variant be started?

#### Recommendations

Cancer surveillance is started promptly after the diagnosis of LFS. However, when performing examination of children, which requires sedation, sufficient considerations need to be given to adverse events such as respiratory and circulatory suppression. The surveillance of adrenocortical cancer begins in childhood, and the surveillance of breast cancer and gastrointestinal cancer begins in adulthood (consensus rate of 100%, 23/23).

#### Explanation

The AACR protocol recommends that cancer surveillance begins immediately after the diagnosis [6]. Some overseas surveillance programs begin once sedation is no longer necessary. When sedation is difficult, examinations and tests can be used in place of surveillance until sedation is no longer necessary. Adrenocortical cancer, brain tumor, and soft tissue sarcoma should be especially observed as they often have childhood onset, but it needs to be known that a surveillance based on examinations and blood has limitations. In terms of sedation, sufficient discussion should be held with the family regarding the possibility of adverse events, such as respiratory and circulatory suppression, and surveillance should be implemented while carefully managing the patient.

In the AACR protocol, since adrenocortical cancer has high penetrance in childhood, US is performed frequently in children aged < 18 years, and surveillance on breast cancer and gastrointestinal cancer begins at 18 (for women) and 25 years, respectively [6]. However, since susceptible age and frequent locations for other LFS core tumors, osteosarcoma, soft tissue sarcoma, and brain tumor (especially glioma), have not been identified, surveillance should begin promptly following the diagnosis of LFS, and should continue over the lifetime of the patient.

## Discussion

Among pediatric patients with cancer, 1–2% are estimated to be individuals with *TP53* pathogenic variant [25–30]. In Japan, about 2,000 children develop cancer every year, 20–40 of them are assumed to have cancer onset due to LFS. According to the present questionnaire survey, only 36 children with LFS were receiving medical care at pediatric cancer facilities in 2017 [117]. Thus, a significant number of patients with LFS might be being overlooked. Alternatively, actual the rate of cancer onset during their lifetime may be lower than assumed, although it is assumed that almost all individuals with *TP53* pathogenic variant are going to develop cancer at least once in their lifetime; thus, the clinical presentation of LFS is still not elucidated. Indeed, the clinical presentation of LFS (types of cancers patients with LFS develop) is affected by molecular biology, such as genotype, but it has been suggested that race also plays a role in these differences. Clarifying the clinical presentation of LFS in Japan will lead to simplification and individualization of health management and cancer surveillance for patients with LFS. With presumption of creating a registration and follow-up system such as registry for individuals with *TP53* pathogenic variant, and providing appropriate follow-up and care, information should be gathered and updated, shared with registrants, and then shared globally.

Patients with LFS are exposed to various environmental factors such as ELSI (Table 5) [118], with some people arguing that a diagnosis of LFS should be proactively avoided. However, since we are entering the era of genomic medicine, the utility of genomic medicine is clear, and diagnosis of LFS is not considered to be a disadvantage. Also, not all of the psychological impacts of LFS are negative [82, 88, 113–116]. Indeed, learning about their genetic background, might make the patient feel motivated to protect their health as well as that of their children and family. We must have a “receptacle” ready to receive these patients with LFS. In other countries, once accurate genetic testing is secured, comprehensive medical management of LFS is steadily being formulated and established. This includes the avoidance of radiation and alkylating agents, cancer prevention and treatment, such as cancer surveillance, measures that lead to early detection of cancer, information update through genetic counseling, and psychological care [6]. In light of such international trends, the option of not diagnosing LFS or not informing the patients of an LFS diagnosis is ethically problematic. Thus, a medical care system needs to be prepared promptly to handle such issues.

The systematic review implemented by our study group did not find any evidence that cancer surveillance would improve the prognosis of individuals with *TP53* pathogenic variant, but the cancer detection rate of cancer surveillance is high, and detected cancers often did not have metastasis. Detecting cancers in the early stage does not equal elimination of cancer death, but detecting the cancer before it invades into surrounding tissues or metastasizes would at least allow for less treatments such, as avoiding radiation therapy, which in turn reduces complications associated with treatments, improves QOL, and leads to prevention of secondary cancer. Prognosis for LFS core sarcomas, such as rhabdomyosarcoma and osteosarcoma are clearly influenced by stage of the cancer at the time of onset, and may be improved as a result of cancer surveillance. Cancer surveillance has various disadvantages, such as false positive, overdiagnosis, and adverse events associated with sedation. However, as the performance of diagnostic imaging machines is rapidly improving with the introduction of AI technology, more accurate and swifter diagnosis will become possible. Furthermore, recent developments in next-generation sequencing technology have improved the precision of molecular biology tests, such as liquid biopsy, which leads to simpler, more accurate, and earlier detection of cancer as an auxiliary test to imaging, or as an alternative test. Given these developments, we can now work to develop a medical care system that will enable cancer surveillance as a management option for patients with LFS. However, the financial burden of genetic tests and surveillance on subjects is enormous. Clinical trials should be publicly subsidized and the results used as the foundation to create a comprehensive treatment system for patients with LFS and hereditary tumor.

## Policy recommendations

In the age of genomic medicine, diagnosis of hereditary tumors such as LFS cannot be avoided. Hereditary tumor diagnosis affects not only the proband but also their close relatives. Clinical trial should be prepared so that those diagnosed with hereditary tumors can participate whenever they need, regardless of the onset of cancer, and based on the result, comprehensive medical care system should be developed. We present following policy recommendations to enable clinical trial implementation:

1. Development of a hereditary tumor registry
2. Development of genetic counselors specialized in hereditary tumors
3. Public subsidies for the cost of genetic tests and cancer surveillance for cancer susceptibility genes such as *TP53*

## Preparation method of the present treatment guidelines

### Preparation of clinical questions (CQs)

We formed a “guideline preparation committee” within our study group, consisting of physicians, genetic counselors, and ethics researchers with expertise in hereditary tumors, and created an analytical framework consisting of medical practices, such as diagnosis of LFS, prevention and treatment, and follow-up, and CQs. For the clinical presentation of LFS, we created CQs outside of the analytical framework. The draft plan for the CQs was prepared based on PICO (P: patients, problem, population, I: interventions, C: comparisons, controls, comparators, O: outcomes), and after thorough discussion involving the whole committee, the guideline preparation committee determined the CQs.

### Systematic review and preparation of the evidence report

We formed a “literature review committee” consisting of physicians who were not experts on hereditary tumors, and were independent of the guideline preparation committee in our study group. These physicians examined literature using PubMed, Embase, and Ichushi-Web, and classified approximately 10,000 papers based on the CQs, conducted abstract review, and full paper review, and determined papers to be evaluated. Furthermore, following data extraction, the evidences were and summarized, and an evidence report was published.

### Recommendations

At the beginning of the study, the aim of the guideline preparation committee was to evaluate advantages and disadvantages based on the evidence report and prepare recommendations for the CQs. However, during the preparation of the evidence report, it became clear that since LFS is a rare disease, there were not many reports that targeted multiple cases, and not many reports that confirmed the standardization and reproducibility of study methods. Furthermore, for many CQs, it was difficult to determine advantages and disadvantages along with the summarization of evidence. Thus, in the present treatment guidelines, we employed expert opinions as needed, while also using the evidence report as much as possible. Whether the recommendation should be consented to or not was determined by votes of the 26 members of the study group consisting of multiple occupations, such as expert physicians of hereditary tumors and genetic counselors, non-expert physicians (pediatricians, pediatric surgeons, radiologists, orthopedic surgeons), ethics researchers, and patient group representative; 23 votes were affirmative. Members of the guideline preparation committee and the literature review committee were excluded from voting. The level of recommendation was not identified since the expert opinions were adopted throughout the guideline; therefore, the consensus rate was calculated from votes and noted.

## External evaluations

The present guidelines were evaluated by the following:

Public comment from The Japanese Society of Pediatric Hematology/Oncology (JSPHO).

Public comment from the Japanese Society for Hereditary Tumors.

As the present guideline adopted expert opinions throughout, it was determined that evaluation by AGREE II would be difficult.

## Revisions

The guideline is to be revised every 3 years (the next revision is scheduled for 2022).

## Research organizations

### Guideline preparation committee

Tadashi Kumamoto: Department of Pediatric Oncology, National Cancer Center Hospital, LFS Expert Group, The Japanese Society for Hereditary Tumors
Yukiko Tsunematsu: Department of Pediatrics, Juntendo University School of Medicine, LFS Expert Group, The Japanese Society for Hereditary Tumors
Yoshiko Nakano: Division of Brain Tumor Translational Research, National Cancer Center Research Institute, Department of Pediatrics, The University of Tokyo Hospital
Hiroyoshi Hattori: Department of Clinical Genetics, National Hospital Organization Nagoya Medical Center
Chieko Tamura: Medical Information and Genetic Counseling Division, FMC Tokyo Clinic, LFS Expert Group, The Japanese Society for Hereditary Tumors
Shimon Tashiro: Department of Sociology, Graduate School of Arts and Letters, Tohoku University

#### Literature review committee

Chisato Hamashima: Department of Nursing, Faculty of Medical Technology, Teikyo University
Teruhiko Terasawa: Department of Emergency and General Internal Medicine, Fujita Health University School of Medicine
Fumito Yamazaki: Department of Pediatrics, Keio University School of Medicine
Takafumi Katayama: Department of Statistics and Information, College of Nursing Art and Science, University of Hyogo

#### Guideline preparation collaborators and consensus voters (not comprehensive)

Akira Nakagawara: Saga International Heavy Ion Cancer Radiation Therapy Center
Yasuhiko Kaneko: Research Institute for Clinical Oncology, Saitama Cancer Center
Shigenobu Suzuki: Department of Ophthalmic Oncology, National Cancer Center Hospital
Akira Kawai: Department of Musculoskeletal Oncology and Rehabilitation Medicine, National Cancer Center Hospital
Tatsuro Tajiri: Department of Pediatric Surgery, Kyoto Prefectural University of Medicine
Atsushi Manabe: Department of Pediatrics, Hokkaido University Graduate School of Medicine
Masatoshi Takagi: Department of Pediatrics and Developmental Biology, Tokyo Medical and Dental University Graduate school of Medical and Dental Science
Mikiko Miyasaka: Department of Radiology, National Center for Child Health and Development
Taiki Nozaki: Department of Radiology, St.Luke's International Hospital
Junko Takita: Department of Pediatrics, Graduate School of Medicine, Kyoto University
Michinori Funato: Department of Pediatrics, National Hospital Organization, Nagara Medical Center
Michiya Ito: Division of Health Administration and Policy, Tohoku Medical and Pharmaceutical University
Naoko Kakee: Division of Bioethics/Division of Information for Specific Pediatric Chronic Diseases, National Center for Child Health and Development
Tomoro Hishiki: Department of Pediatric Surgery, Chiba University Graduate School of Medicine
Shinsuke Hirabayashi: Department of Pediatrics, Hokkaido University Graduate School of Medicine
Tomoaki Mori: Department of Musculoskeletal Oncology and Rehabilitation, National Cancer Center Hospital
Akihiro Sakurai: Department of Medical Genetics and Genomics, Sapporo Medical University School of Medicine
Katsunori Fujii: Department of Pediatrics, Chiba University Graduate School of Medicine
Hiroshi Yagata (deceased): Department of Breast Care, Saitama Medical Center
Akira Shimada: Department of Pediatric Hematology/Oncology, Okayama University Hospital
Eizou Hiyama: Department of Pediatric Surgery, Hiroshima University Hospital
Hideki Muramatsu: Department of Pediatrics, Nagoya University Graduate School of Medicine
Masahiro Yao: Department of Urology, Yokohama City University Graduate School of Medicine
Masayoshi Tsutsumi: Japan Registered Clinical Laboratories Association
Naonori Kawakubo: Department of pediatric surgery, faculty of medical sciences, Kyushu University
Teruhiko Yoshida: Department of Genetic Medicine and Service, National Cancer Center Hospital
Takeshi Nakajima: Department of Clinical Genetics, Cancer Institute Hospital
Yoshiko Nakayama: Department of Pediatrics, Shinshu University School of Medicine
Akiko Higuchi: Children’s Cancer Association of Japan
Hiroko Kondo: Children’s Cancer Association of Japan
Asako Katayama: Children’s Cancer Association of Japan

## Abbreviations

AACR: American Association of Cancer Research
AYA: Adolescent and young adult
ELSI: Ethical, legal, and social issues
GI: Gastro-intestinal
HR: Hezard ratio
LFS: Li–Fraumeni syndrome
NCCN: National Comprehensive Cancer Network
NCI: National Cancer Institute
NGS: Next-generation sequencing
OR: Odds ratio
QOL: Quality of life
RR: Relative risk
SHH: Sonic hedge hog
SIR: Standardized incidence ratio
US: Ultrasonography
WB MRI: Whole-body MRI
