# Correction to: Medical guidelines for Li–Fraumeni syndrome 2019, version 1.1

**DOI:** 10.1007/s10147-021-02086-5

**Published:** 2021-12-03

**Authors:** Tadashi Kumamoto, Fumito Yamazaki, Yoshiko Nakano, Chieko Tamura, Shimon Tashiro, Hiroyoshi Hattori, Akira Nakagawara, Yukiko Tsunematsu

**Affiliations:** 1 Department of Pediatric Oncology, National Cancer Center Hospital, Tokyo, Japan; 2 Department of Pediatrics, Keio University School of Medicine, Tokyo, Japan; 3 Division of Brain Tumor Translational Research, National Cancer Center Research Institute, Tokyo, Japan; 4 Department of Pediatrics, The University of Tokyo Hospital, Tokyo, Japan; 5 Medical Information and Genetic Counseling Division, FMC Tokyo Clinic, Tokyo, Japan; 6 Department of Sociology, Graduate School of Arts and Letters, Tohoku University, Sendai, Japan; 7 Department of Clinical Genetics, National Hospital Organization Nagoya Medical Center, Aichi, Japan; 8 Saga International Heavy Ion Cancer Radiation Therapy Center, Saga, Japan

## Correction to: International Journal of Clinical Oncology (2021) 26:2161–2178 10.1007/s10147-021-02011-w

The article Medical guidelines for Li–Fraumeni syndrome 2019, version 1.1 written by Tadashi Kumamoto, Fumito Yamazaki, Yoshiko Nakano, Chieko Tamura, Shimon Tashiro, Hiroyoshi Hattori, Akira Nakagawara and Yukiko Tsunematsu was originally published Online First without Open Access.

With the author(s)’ decision to opt for Open Choice the copyright of the article changed on November 19, 2021 to © Author(s) 2021 and the article is forthwith distributed under a Creative Commons Attribution 4.0 International License, which permits use, sharing, adaptation, distribution and reproduction in any medium or format, as long as you give appropriate credit to the original author(s) and the source, provide a link to the Creative Commons licence, and indicate if changes were made. The images or other third party material in this article are included in the article’s Creative Commons licence, unless indicated otherwise in a credit line to the material. If material is not included in the article’s Creative Commons licence and your intended use is not permitted by statutory regulation or exceeds the permitted use, you will need to obtain permission directly from the copyright holder. To view a copy of this licence, visit http://creativecommons.org/licenses/by/4.0.

The original article has been corrected.

